# 4-Benzyl-3-[(1-oxidoethylidene)amino]-1-phenyl-4,5-dihydro-1*H*-1,2,4-triazol-5-iminium

**DOI:** 10.1107/S1600536811006751

**Published:** 2011-03-12

**Authors:** Victor M. Chernyshev, Anna G. Mazharova, Victor B. Rybakov

**Affiliations:** a South-Russia State Technical University, 346428 Novocherkassk, Russian Federation; b Department of Chemistry, Moscow State University, 119992 Moscow, Russian Federation

## Abstract

The title compound, C_17_H_17_N_5_O, exists in the zwitterionic form with the amide group deprotonated. The mean planes of the 1,2,4-triazole and *N*-phenyl rings form a dihedral angle of 39.14 (8)°. The N atom of the amino group adopts a trigonal configuration. Inter­moleculat C—H⋯O and C—H⋯N hydrogen bonds occur. In the crystal, mol­ecules are linked into a two-dimensional network parallel to (10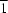) by N—H⋯O and N—H⋯N hydrogen bonds. C—H⋯N contacts are also observed.

## Related literature

For the synthesis of the starting compound, *N*-(5-amino-1-phenyl-1*H*-1,2,4-triazol-3-yl)acetamide, see: Chernyshev *et al.* (2005 ▶). For alkyl­ation and other reactions of related compounds with electrophiles, see: Chernyshev *et al.* (2008*a* ▶,*b* ▶). For crystal structures of 3(5)-acyl­amino-1,2,4-triazoles, see: Selby & Lepone (1984 ▶); Gyorgydeak *et al.* (1995 ▶); Chernyshev *et al.* (2006 ▶); Masiukiewicz *et al.* (2007 ▶); Miao *et al.* (2009 ▶). For crystal structures of 5-amino-1,2,4-triazolium salts, see: Darwich *et al.* (2008*a* ▶,*b* ▶); Klapotke & Sabate (2008 ▶); Tao *et al.* (2009 ▶); Chernyshev *et al.* (2010 ▶). For standard bond lengths, see: Allen *et al.* (1987 ▶). For the correlation of bond lengths with bond orders in *sp* ^2^-hybridized C and N atoms, see: Burke-Laing & Laing (1976 ▶).
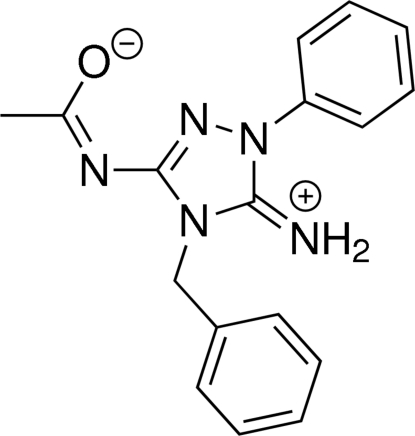


## Experimental

### 

#### Crystal data

C_17_H_17_N_5_O
*M* *_r_* = 307.36
Monoclinic, 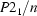

*a* = 10.262 (2) Å
*b* = 15.240 (3) Å
*c* = 10.967 (2) Å
β = 113.86 (2)°
*V* = 1568.6 (6) Å^3^
*Z* = 4
Ag *K*α radiation
λ = 0.56085 Å
μ = 0.06 mm^−1^
*T* = 295 K
0.20 × 0.20 × 0.20 mm

#### Data collection

Enraf–Nonius CAD-4 diffractometer
3582 measured reflections
3412 independent reflections
2294 reflections with *I* > 2σ(*I*)
*R*_int_ = 0.068
1 standard reflections every 6 min  intensity decay: 0%

#### Refinement

*R*[*F* ^2^ > 2σ(*F* ^2^)] = 0.059
*wR*(*F* ^2^) = 0.160
*S* = 1.03
3412 reflections
218 parameters
H atoms treated by a mixture of independent and constrained refinement
Δρ_max_ = 0.30 e Å^−3^
Δρ_min_ = −0.22 e Å^−3^

### 

Data collection: *CAD-4 EXPRESS* (Enraf–Nonius, 1994 ▶); cell refinement: *CAD-4 EXPRESS*; data reduction: *XCAD4* (Harms & Wocadlo, 1995 ▶); program(s) used to solve structure: *SHELXS97* (Sheldrick, 2008 ▶); program(s) used to refine structure: *SHELXL97* (Sheldrick, 2008 ▶); molecular graphics: *ORTEP-3* (Farrugia, 1997 ▶); software used to prepare material for publication: *WinGX* (Farrugia, 1999 ▶).

## Supplementary Material

Crystal structure: contains datablocks global, I. DOI: 10.1107/S1600536811006751/aa2002sup1.cif

Structure factors: contains datablocks I. DOI: 10.1107/S1600536811006751/aa2002Isup2.hkl

Additional supplementary materials:  crystallographic information; 3D view; checkCIF report

## Figures and Tables

**Table 1:** Hydrogen-bond geometry (Å, °)

*D*—H⋯*A*	*D*—H	H⋯*A*	*D*⋯*A*	*D*—H⋯*A*
N51—H51*A*⋯O14^i^	0.94 (3)	1.83 (3)	2.739 (3)	161 (2)
N51—H51*B*⋯N13^ii^	0.84 (3)	2.18 (3)	2.971 (3)	157 (3)
C6—H6*B*⋯O14	0.97	2.30	3.045 (3)	133
C8—H8⋯N13	0.93	2.72	3.454 (4)	136
C12—H12⋯N13^ii^	0.93	2.54	3.441 (4)	162

Symmetry codes: (i) 
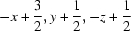
; (ii) 
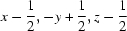
.

**Fig. 1.**
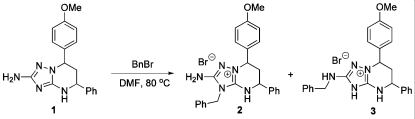
Alkylation of the compound 1 with benzyl bromide (Chernyshev et al., 2008a).

**Fig. 2.**
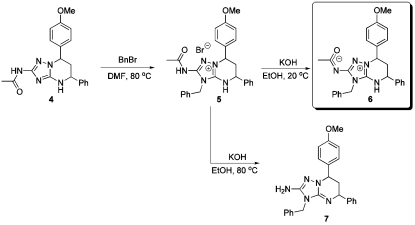
Regioselective alkylation of the compound 4 with benzyl bromide and structure of the compound 6 (Chernyshev et al., 2008a).

**Fig. 3.**
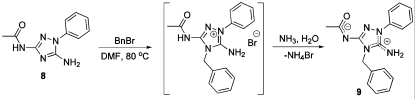
Synthesis of the compound 9.

**Fig. 4.**
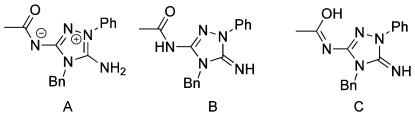
The possible tautomeric forms of the compound 9.

**Fig. 5.**
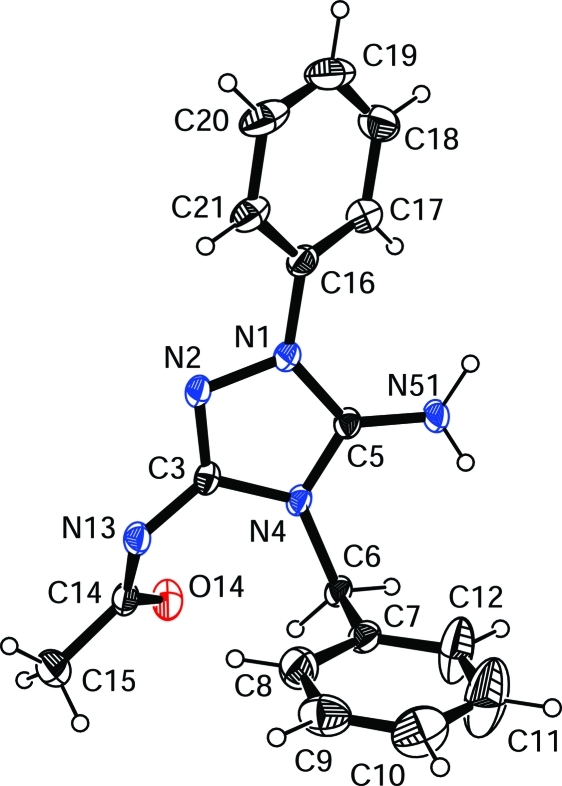
ORTEP-3 (Farrugia, 1997) plot of molecular structure of the title compound showing the atom-numbering scheme. Displacement ellipsoids are drawn at the 30% probability level. H atoms are presented as a small spheres of arbitrary radius.

**Fig. 6.**
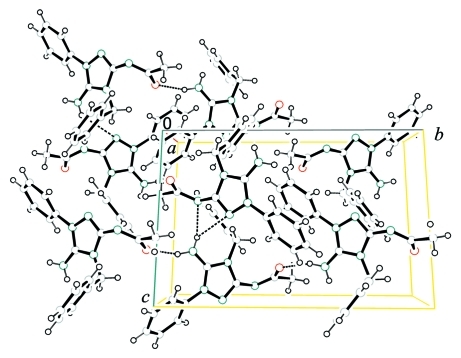
The crystal packing of the title compound along the b axis. Hydrogen bonds are shown as dashed lines.

**Fig. 7.**
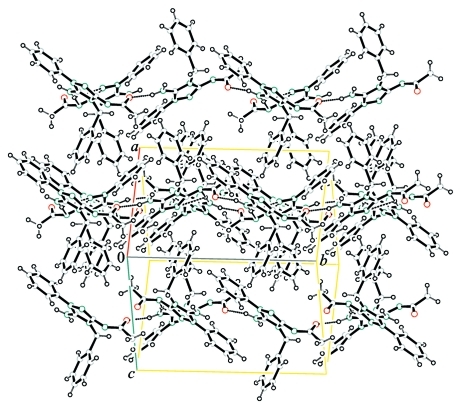
The crystal packing of the title compound along the [1, 0, -1] plane, showing two-dimensional net of hydrogen bonds which are drawn as dashed lines.

## supplementary crystallographic information

### Comment

Previously, during investigation of the reactions of 2-amino-4,5,6,7-tetrahydro-1,2,4-triazolo-[1,5-*a*]pyrimidines (Chernyshev *et al.*, 2008*a*) we revealed that quaternization of the 2-amino-7-(4-methoxyphenyl)-5-phenyl-4,5,6,7-tetrahydro-1,2,4- triazolo[1,5-*a*]pyrimidine (1) with benzyl bromide occurred nonselectively and afforded a mixture of compounds 2 and 3 (Fig. 1). Selective alkylation was possible only after protection of the amino group of compound 1 by acylation (Fig. 2). As a result of quaternization of the compound 4 we obtained bromide 5, which was converted into the compound 6 under the action of KOH at room temperature and into the compound 7 at heating. The zwitterionic structure of the compound 6 was proposed on the basis of indirect data, *i.e.* comparison of its acid–base properties and spectral characteristics with the compound 7, which was considered as the fixed inverse tautomeric form. Unfortunately, we could not confirm the structure of the compound 6 by X-ray analysis due to difficulties in growing a suitable crystal. It was demonstrated in our previous works (Chernyshev *et al.*, 2008**a*,b*) that the chemical properties of 2-amino-4,5,6,7-tetrahydro-1,2,4-triazolo-[1,5-*a*]pyrimidines in many respects are analogous to 1-substituted 3,5-diamino-1,2,4-triazoles. For elaboration of a selective method for the preparation of 1,4-disubstituted 3,5-diamino-1,2,4-triazoles and additional confirmation of the structure of compound 6, we investigated the alkylation of *N*-(5-amino-1-phenyl-1*H*-1,2,4-triazol-3-yl)acetamide (8) (Fig. 3). The present report describes our results of the X-ray investigation of the structure of compound 9, which can be considered as a structural analog of the compound 6.

The possibility of existence of the three tautomeric forms A–B can be presumed for the compound 9 (Fig. 4). In accordance with the X-ray diffraction data, the studied compound in the crystal exists as the zwitterionic tautomer A (Fig. 5). The triazole ring is planar, with the mean deviations of the ring atoms from their least-squares plane being 0.01 (2) Å. The *N*-phenyl and triazole rings are essentially noncoplanar, with a dihedral angle of 39.14 (8)°. The atom N13 of the deprotonated amide group deviates from the least-squares plane of triazole ring by 0.158 (2) Å, the dihedral angle between the planes of the amide group (N13/C14/O14) and triazole cycle amounts 54.6 (2)°. The length of the bond N13—C14 (1.333 (3) Å) is close to the length of the double bond Nsp^2^—C*sp*^2^ (Allen *et al.*, 1987), whereas the bond O14—C14 (1.251 (2) Å) is longer then the typical amide bond (1.234 Å) (Allen *et al.*, 1987). These results indicate a pronounced delocalization of bonds and negative charge in the deprotonated CON fragment. The bond N13—C3 (1.359 (3) Å) is slightly shorter than the analogous bond in the unionized 3-acylamino-1,2,4-triazoles (1.381 Å–1.395 Å) (Selby & Lepone, 1984; Gyorgydeak *et al.*, 1995; Chernyshev *et al.*, 2006; Masiukiewicz *et al.*, 2007; Miao *et al.*, 2009), probably as a result of an attractive polar interaction between the opposite charged amide and triazole fragments. The N51 atom deviates from the plane of the triazole ring by 0.060 (2) Å. Amino grope adopts a plane configuration (the sum of valence angles is 359.6°) and almost coplanar with the triazole cycle, forming a dihedral angle of 9.1 (2)°. The bonds N4—C5 and N1—C5 of the triazole cycle have almost equal length (1.342 (2)Å and 1.347 (3) Å, correspondingly), however the bond C5—N4 (1.313 (3) Å) is considerably shorter in relation to a purely single Nsp^2^—C*sp*^2^ bond (1.43 Å–1.45 Å) (Burke-Laing & Laing, 1976). It indicates the delocalization of the positive charge in the fragment N4/C5/N51/N1 and the considerable contribution of the imino form to the molecular structure of the discussed compound, analogously to another 5-amino-1,2,4-triazolium salts (Darwich *et al.*, 2008**a*,b*; Klapotke & Sabate, 2008; Tao *et al.*, 2009; Chernyshev *et al.*, 2010).

Two classical intermolecular hydrogen bonds are found in crystal structure (Table 1): N51—H51A···O14^i^ with parameters - N51—H21A^i^ = 0.94 (3) Å, H51A···O14 = 1.83 (3) Å, N51···O14^i^ = 2.739 (3)Å and angle N51—H21A···O14^i^ = 160 (3)°; N51—H21B···N13^ii^ with parameters - N51—H21B^ii^ = 0.84 (3) Å, H51B···N13^ii^ = 2.18 (3) Å, N51···N13^ii^ = 2.971 (3)Å and angle N51—H21B···N13^ii^ = 157 (3)°. Four non-classical hydrogen bonds are found in crystal structure (Table 1): C6—H6B···O14 with parameters - C6—H6B = 0.97 Å, H6B···O14 = 2.30 Å, C6···O14 = 3.045 (3)Å and angle C6—H6B···O14 = 133°; C8—H8···N13 with parameters - C8—H8 = 0.93 Å, H8···N13 = 2.72 Å, C8···N13 = 3.454 (4)Å and angle C8—H8···N13 = 136°; C17—H17···N51 with parameters - C17—H17 = 0.93 Å, H17···N51 = 2.75 Å, C17···N51 = 3.146 (4)Å and angle C17—H17···N21 = 107°; C12—H12···N13^ii^ with parameters - C12—H12 = 0.93 Å, H12···N13^ii^ = 2.54 Å, C12···N13^ii^ = 3.441 (4)Å and angle C12—H12···N13^ii^ = 162°. Symmetry codes: (i) -*x* + 3/2, *y* + 1/2, -*z* + 1/2; (ii) *x* - 1/2, -*y* + 1/2, *z* - 1/2.

Due to the structural similarity of compounds 6 and 9, we can conclude that the present results corroborate our previous deduction on the zwitterionic structure of compound 6.

### Experimental

The crystals of the acetyl(5-amino-4-benzyl-1-phenyl-4*H*-1,2,4-triazol-1-ium-3 -yl)azanide (9) suitable for X-ray analysis were grown by slow evaporation of ethanol solution at room temperature within one week. The title compound was prepared by the following procedure.

A mixture of compound 8 (2 g, 9.2 mmol), benzyl bromide (1.89 g, 11.1 mmol) and *DMF* (4.0 ml) was heated at 353 K and stirring for 4 h, then cooled to room temperature and diluted with 20% aqueous solution of NH_3_ (8 ml). The resulted mixture was cooled to 276–278 K and the precipitate formed was isolated by filtration, washed with cold water, recrystallized from ethanol and dried at 373 K to give 2.21 g (78% yield) of compound 9. White powder, m. p. 472–473 K. Spectrum ^13^C NMR (150 MHz), *δ*: 22.57 (CH_3_), 43.99 (CH_2_), 118.54, 123.93, 127.12, 127.54, 128.54, 128.79, 135.86, 138.75 (carbons of phenyls), 141.16, 150.43 (carbons of triazole), 170.64 (CO). MS (EI, 70 eV), *m*/*z* (%): 307 (6) [*M*^+^], 265 (8), 119 (7), 104 (11), 91 (100), 77 (31), 65 (18), 43 (48). Anal. Calcd for C_17_H_17_N_5_O: C, 66.43; H, 5.58; N, 22.79. Found: C, 66.27; H, 5.49; N, 22.98.

The starting *N*-(5-amino-1-phenyl-1*H*-1,2,4-triazol-3-yl)acetamide (8) was obtained by the known method (Chernyshev *et al.*, 2005).

### Refinement

C-bound H atoms were placed in calculated positions C—H 0.93Å for aromatic, C—H = 0.97Å for CH_2_, C—H = 0.96Å for CH_3_ and refined as riding, with *U*_iso_(H) = 1.2(1.5)*U*_eq_(C). H-atoms forming hydrogen (N-bound H atoms) bonds were found from difference Fourier map and refined independently.

### Crystal data

**Table:**

C_17_H_17_N_5_O	*F*(000) = 648
*M**_r_* = 307.36	*D*_x_ = 1.301 Mg m^−^^3^
Monoclinic, *P*2_1_/*n*	Melting point = 472–473 K
Hall symbol: -P 2yn	Ag *K*α radiation, λ = 0.56085 Å
*a* = 10.262 (2) Å	Cell parameters from 25 reflections
*b* = 15.240 (3) Å	θ = 13.2–14.4°
*c* = 10.967 (2) Å	µ = 0.06 mm^−^^1^
β = 113.86 (2)°	*T* = 295 K
*V* = 1568.6 (6) Å^3^	Prism, colourless
*Z* = 4	0.20 × 0.20 × 0.20 mm

### Data collection

**Table:**

Enraf–Nonius CAD-4 diffractometer	*R*_int_ = 0.068
Radiation source: fine-focus sealed tube	θ_max_ = 21.0°, θ_min_ = 1.8°
graphite	*h* = −13→11
Non–profiled ω scans	*k* = 0→19
3582 measured reflections	*l* = 0→13
3412 independent reflections	1 standard reflections every 6 min
2294 reflections with *I* > 2σ(*I*)	intensity decay: −1%

### Refinement

**Table:**

Refinement on *F*^2^	Secondary atom site location: difference Fourier map
Least-squares matrix: full	Hydrogen site location: inferred from neighbouring sites
*R*[*F*^2^ > 2σ(*F*^2^)] = 0.059	H atoms treated by a mixture of independent and constrained refinement
*wR*(*F*^2^) = 0.160	*w* = 1/[σ^2^(*F*_o_^2^) + (0.0799*P*)^2^ + 0.4611*P*] where *P* = (*F*_o_^2^ + 2*F*_c_^2^)/3
*S* = 1.03	(Δ/σ)_max_ < 0.001
3412 reflections	Δρ_max_ = 0.30 e Å^−^^3^
218 parameters	Δρ_min_ = −0.22 e Å^−^^3^
0 restraints	Extinction correction: *SHELXL97* (Sheldrick, 2008), Fc^*^=kFc[1+0.001xFc^2^λ^3^/sin(2θ)]^-1/4^
Primary atom site location: structure-invariant direct methods	Extinction coefficient: 0.066 (6)

### Special details

**Table:**

Geometry. All s.u.'s (except the s.u. in the dihedral angle between two l.s. planes) are estimated using the full covariance matrix. The cell s.u.'s are taken into account individually in the estimation of s.u.'s in distances, angles and torsion angles; correlations between s.u.'s in cell parameters are only used when they are defined by crystal symmetry. An approximate (isotropic) treatment of cell s.u.'s is used for estimating s.u.'s involving l.s. planes.
Refinement. Refinement of F^2^ against ALL reflections. The weighted R-factor wR and goodness of fit S are based on F^2^, conventional R-factors R are based on F, with F set to zero for negative F^2^. The threshold expression of F^2^ > 2σ(F^2^) is used only for calculating R-factors(gt) etc. and is not relevant to the choice of reflections for refinement. R-factors based on F^2^ are statistically about twice as large as those based on F, and R-factors based on ALL data will be even larger.

### Fractional atomic coordinates and isotropic or equivalent isotropic displacement parameters (Å^2^)

**Table:**

	*x*	*y*	*z*	*U*_iso_*/*U*_eq_	
N1	0.86753 (18)	0.33588 (11)	0.43051 (16)	0.0345 (4)	
N2	0.96176 (18)	0.26764 (11)	0.49396 (16)	0.0355 (4)	
C3	0.9547 (2)	0.21474 (13)	0.39830 (19)	0.0314 (5)	
N4	0.85963 (17)	0.24731 (11)	0.27474 (15)	0.0314 (4)	
C5	0.8081 (2)	0.32385 (13)	0.29796 (19)	0.0315 (5)	
C6	0.8381 (2)	0.21434 (14)	0.14185 (19)	0.0340 (5)	
H6A	0.7417	0.2280	0.0797	0.041*	
H6B	0.8481	0.1510	0.1459	0.041*	
C7	0.9406 (2)	0.25232 (14)	0.0901 (2)	0.0383 (5)	
C8	1.0832 (3)	0.2325 (2)	0.1442 (3)	0.0597 (7)	
H8	1.1190	0.1948	0.2169	0.072*	
C9	1.1747 (3)	0.2673 (2)	0.0931 (3)	0.0688 (8)	
H9	1.2711	0.2533	0.1322	0.083*	
C10	1.1255 (4)	0.3211 (2)	−0.0126 (4)	0.0812 (10)	
H10	1.1868	0.3434	−0.0484	0.097*	
C11	0.9848 (5)	0.3428 (3)	−0.0668 (5)	0.128 (2)	
H11	0.9499	0.3807	−0.1395	0.153*	
C12	0.8939 (3)	0.3090 (3)	−0.0146 (4)	0.0911 (12)	
H12	0.7983	0.3252	−0.0518	0.109*	
N13	1.04305 (18)	0.14482 (11)	0.41734 (17)	0.0371 (4)	
C14	0.9911 (2)	0.06725 (14)	0.3633 (2)	0.0393 (5)	
O14	0.86200 (18)	0.04827 (10)	0.30395 (16)	0.0492 (5)	
C15	1.1022 (3)	−0.00100 (17)	0.3782 (3)	0.0563 (7)	
H15A	1.0685	−0.0575	0.3914	0.084*	
H15B	1.1885	0.0131	0.4536	0.084*	
H15C	1.1207	−0.0022	0.2991	0.084*	
C16	0.8529 (2)	0.40728 (14)	0.5074 (2)	0.0381 (5)	
C17	0.7220 (3)	0.44477 (17)	0.4786 (2)	0.0520 (6)	
H17	0.6411	0.4231	0.4095	0.062*	
C18	0.7124 (4)	0.5152 (2)	0.5538 (3)	0.0678 (8)	
H18	0.6249	0.5424	0.5333	0.081*	
C19	0.8311 (5)	0.5452 (2)	0.6586 (3)	0.0751 (10)	
H19	0.8238	0.5924	0.7092	0.090*	
C20	0.9602 (4)	0.50577 (19)	0.6889 (3)	0.0637 (8)	
H20	1.0397	0.5256	0.7614	0.076*	
C21	0.9741 (3)	0.43706 (16)	0.6133 (2)	0.0474 (6)	
H21	1.0624	0.4112	0.6327	0.057*	
N51	0.7197 (2)	0.37631 (13)	0.2071 (2)	0.0438 (5)	
H51A	0.695 (3)	0.433 (2)	0.223 (2)	0.053 (7)*	
H51B	0.689 (3)	0.361 (2)	0.127 (3)	0.070 (9)*	

### Atomic displacement parameters (Å^2^)

**Table:**

	*U*^11^	*U*^22^	*U*^33^	*U*^12^	*U*^13^	*U*^23^
N1	0.0351 (9)	0.0355 (9)	0.0252 (9)	0.0034 (7)	0.0041 (7)	−0.0009 (7)
N2	0.0372 (10)	0.0353 (10)	0.0254 (8)	0.0043 (7)	0.0036 (7)	0.0026 (7)
C3	0.0282 (10)	0.0337 (10)	0.0258 (10)	−0.0020 (8)	0.0040 (8)	0.0037 (8)
N4	0.0315 (9)	0.0324 (9)	0.0220 (8)	−0.0001 (7)	0.0022 (7)	0.0010 (7)
C5	0.0309 (10)	0.0290 (10)	0.0269 (10)	−0.0015 (8)	0.0036 (8)	−0.0007 (8)
C6	0.0371 (11)	0.0330 (11)	0.0229 (9)	−0.0005 (9)	0.0029 (8)	−0.0017 (8)
C7	0.0432 (12)	0.0380 (11)	0.0307 (11)	−0.0021 (10)	0.0119 (9)	−0.0026 (9)
C8	0.0503 (16)	0.0741 (19)	0.0557 (16)	0.0122 (13)	0.0223 (13)	0.0085 (14)
C9	0.0537 (17)	0.083 (2)	0.079 (2)	−0.0007 (15)	0.0357 (16)	−0.0137 (18)
C10	0.082 (2)	0.092 (2)	0.088 (2)	−0.021 (2)	0.053 (2)	0.001 (2)
C11	0.085 (3)	0.190 (5)	0.111 (3)	0.003 (3)	0.043 (2)	0.089 (4)
C12	0.0585 (19)	0.130 (3)	0.080 (2)	0.0083 (19)	0.0224 (17)	0.063 (2)
N13	0.0355 (9)	0.0360 (10)	0.0298 (9)	0.0032 (8)	0.0029 (7)	0.0018 (8)
C14	0.0459 (13)	0.0349 (11)	0.0293 (11)	0.0040 (10)	0.0071 (9)	0.0053 (9)
O14	0.0477 (10)	0.0357 (8)	0.0474 (10)	−0.0052 (7)	0.0019 (7)	0.0030 (7)
C15	0.0608 (16)	0.0463 (14)	0.0544 (16)	0.0123 (12)	0.0158 (13)	−0.0009 (12)
C16	0.0493 (13)	0.0356 (11)	0.0290 (11)	−0.0031 (10)	0.0154 (9)	0.0011 (9)
C17	0.0600 (16)	0.0531 (15)	0.0425 (13)	0.0068 (12)	0.0202 (12)	−0.0015 (11)
C18	0.096 (2)	0.0560 (17)	0.0662 (19)	0.0191 (16)	0.0479 (18)	0.0045 (15)
C19	0.135 (3)	0.0474 (16)	0.0628 (19)	−0.0084 (19)	0.061 (2)	−0.0137 (14)
C20	0.099 (2)	0.0552 (16)	0.0436 (15)	−0.0323 (17)	0.0357 (15)	−0.0163 (12)
C21	0.0587 (15)	0.0486 (13)	0.0332 (12)	−0.0147 (11)	0.0167 (11)	−0.0057 (10)
N51	0.0510 (12)	0.0332 (10)	0.0284 (10)	0.0102 (9)	−0.0033 (8)	−0.0023 (8)

### Geometric parameters (Å, °)

**Table:**

N4—C5	1.347 (3)	C11—H11	0.9300
N4—C3	1.402 (2)	C12—H12	0.9300
N4—C6	1.471 (3)	N13—C14	1.333 (3)
C5—N51	1.313 (3)	C14—O14	1.251 (3)
C5—N1	1.342 (2)	C14—C15	1.503 (3)
N1—N2	1.399 (2)	C15—H15A	0.9600
N1—C16	1.422 (3)	C15—H15B	0.9600
N2—C3	1.302 (3)	C15—H15C	0.9600
C3—N13	1.359 (3)	C16—C17	1.374 (3)
C6—C7	1.499 (3)	C16—C21	1.390 (3)
C6—H6A	0.9700	C17—C18	1.382 (4)
C6—H6B	0.9700	C17—H17	0.9300
C7—C12	1.360 (4)	C18—C19	1.371 (5)
C7—C8	1.371 (3)	C18—H18	0.9300
C8—C9	1.379 (4)	C19—C20	1.368 (5)
C8—H8	0.9300	C19—H19	0.9300
C9—C10	1.341 (5)	C20—C21	1.378 (4)
C9—H9	0.9300	C20—H20	0.9300
C10—C11	1.361 (5)	C21—H21	0.9300
C10—H10	0.9300	N51—H51A	0.94 (3)
C11—C12	1.378 (5)	N51—H51B	0.84 (3)
			
C5—N4—C3	107.27 (16)	C7—C12—C11	121.5 (3)
C5—N4—C6	124.75 (16)	C7—C12—H12	119.3
C3—N4—C6	127.11 (17)	C11—C12—H12	119.3
N51—C5—N1	127.6 (2)	C14—N13—C3	120.35 (18)
N51—C5—N4	125.98 (19)	O14—C14—N13	125.8 (2)
N1—C5—N4	106.38 (16)	O14—C14—C15	119.6 (2)
C5—N1—N2	110.92 (16)	N13—C14—C15	114.6 (2)
C5—N1—C16	129.54 (17)	C14—C15—H15A	109.5
N2—N1—C16	119.44 (16)	C14—C15—H15B	109.5
C3—N2—N1	104.94 (15)	H15A—C15—H15B	109.5
N2—C3—N13	123.07 (17)	C14—C15—H15C	109.5
N2—C3—N4	110.47 (17)	H15A—C15—H15C	109.5
N13—C3—N4	125.89 (18)	H15B—C15—H15C	109.5
N4—C6—C7	113.32 (17)	C17—C16—C21	121.2 (2)
N4—C6—H6A	108.9	C17—C16—N1	120.7 (2)
C7—C6—H6A	108.9	C21—C16—N1	118.2 (2)
N4—C6—H6B	108.9	C16—C17—C18	119.0 (3)
C7—C6—H6B	108.9	C16—C17—H17	120.5
H6A—C6—H6B	107.7	C18—C17—H17	120.5
C12—C7—C8	117.1 (2)	C19—C18—C17	120.3 (3)
C12—C7—C6	120.2 (2)	C19—C18—H18	119.8
C8—C7—C6	122.7 (2)	C17—C18—H18	119.8
C7—C8—C9	121.5 (3)	C20—C19—C18	120.2 (3)
C7—C8—H8	119.2	C20—C19—H19	119.9
C9—C8—H8	119.2	C18—C19—H19	119.9
C10—C9—C8	120.4 (3)	C19—C20—C21	120.9 (3)
C10—C9—H9	119.8	C19—C20—H20	119.6
C8—C9—H9	119.8	C21—C20—H20	119.6
C9—C10—C11	119.3 (3)	C20—C21—C16	118.4 (3)
C9—C10—H10	120.4	C20—C21—H21	120.8
C11—C10—H10	120.4	C16—C21—H21	120.8
C10—C11—C12	120.3 (3)	C5—N51—H51A	125.1 (15)
C10—C11—H11	119.9	C5—N51—H51B	118 (2)
C12—C11—H11	119.9	H51A—N51—H51B	116 (3)
			
C3—N4—C5—N51	177.4 (2)	C7—C8—C9—C10	−0.7 (5)
C6—N4—C5—N51	7.4 (3)	C8—C9—C10—C11	1.6 (6)
C3—N4—C5—N1	−1.2 (2)	C9—C10—C11—C12	−0.8 (7)
C6—N4—C5—N1	−171.22 (18)	C8—C7—C12—C11	2.0 (6)
N51—C5—N1—N2	−177.0 (2)	C6—C7—C12—C11	−178.0 (4)
N4—C5—N1—N2	1.6 (2)	C10—C11—C12—C7	−1.1 (8)
N51—C5—N1—C16	−0.7 (4)	N2—C3—N13—C14	135.3 (2)
N4—C5—N1—C16	177.9 (2)	N4—C3—N13—C14	−54.2 (3)
C5—N1—N2—C3	−1.4 (2)	C3—N13—C14—O14	−7.3 (3)
C16—N1—N2—C3	−178.07 (18)	C3—N13—C14—C15	172.8 (2)
N1—N2—C3—N13	172.37 (18)	C5—N1—C16—C17	42.3 (3)
N1—N2—C3—N4	0.5 (2)	N2—N1—C16—C17	−141.7 (2)
C5—N4—C3—N2	0.4 (2)	C5—N1—C16—C21	−138.6 (2)
C6—N4—C3—N2	170.09 (18)	N2—N1—C16—C21	37.4 (3)
C5—N4—C3—N13	−171.11 (19)	C21—C16—C17—C18	2.2 (4)
C6—N4—C3—N13	−1.4 (3)	N1—C16—C17—C18	−178.7 (2)
C5—N4—C6—C7	82.4 (2)	C16—C17—C18—C19	−2.2 (4)
C3—N4—C6—C7	−85.5 (2)	C17—C18—C19—C20	0.4 (5)
N4—C6—C7—C12	−110.7 (3)	C18—C19—C20—C21	1.5 (4)
N4—C6—C7—C8	69.3 (3)	C19—C20—C21—C16	−1.5 (4)
C12—C7—C8—C9	−1.1 (4)	C17—C16—C21—C20	−0.4 (3)
C6—C7—C8—C9	178.9 (2)	N1—C16—C21—C20	−179.5 (2)

### Hydrogen-bond geometry (Å, °)

**Table:**

*D*—H···*A*	*D*—H	H···*A*	*D*···*A*	*D*—H···*A*
N51—H51A···O14^i^	0.94 (3)	1.83 (3)	2.739 (3)	161 (2)
N51—H51B···N13^ii^	0.84 (3)	2.18 (3)	2.971 (3)	157 (3)
C6—H6B···O14	0.97	2.30	3.045 (3)	133
C8—H8···N13	0.93	2.72	3.454 (4)	136
C17—H17···N51	0.93	2.75	3.146 (4)	107
C12—H12···N13^ii^	0.93	2.54	3.441 (4)	162

Symmetry codes: (i) −*x*+3/2, *y*+1/2, −*z*+1/2; (ii) *x*−1/2, −*y*+1/2, *z*−1/2.

### Table 2 Comparitive selected parameters (Å) for estimated bond distances and corrected following Busing &amp; Levy, (1964).

**Table:**

Bond	Uncorrected	Lower bound	Upper bound	Riding motion	Non–correlated motion
C6–C7	1.499 (3)	1.499	1.583	1.504	1.541
C7–C8	1.371 (3)	1.375	1.517	1.394	1.446
C7–C12	1.360 (4)	1.375	1.575	1.418	1.475
C8–C9	1.379 (4)	1.380	1.573	1.388	1.476
C9–C10	1.341 (5)	1.342	1.539	1.356	1.440
C10–C11	1.361 (5)	1.370	1.679	1.413	1.524
C11–C12	1.378 (5)	1.382	1.773	1.417	1.577
N13–C14	1.333 (3)	1.333	1.449	1.335	1.391
C14–O14	1.251 (3)	1.252	1.393	1.264	1.323
C14–C15	1.503 (3)	1.505	1.636	1.519	1.570
C16–C17	1.374 (3)	1.375	1.493	1.388	1.434
C16–C21	1.390 (3)	1.391	1.519	1.401	1.455
C17–C18	1.382 (4)	1.383	1.573	1.397	1.478
C18–C19	1.371 (5)	1.372	1.538	1.378	1.455
C19–C20	1.368 (5)	1.369	1.500	1.379	1.434
C20–C21	1.378 (4)	1.380	1.549	1.394	1.464
